# Bilateral upper eyelid swelling (Hoagland sign) in Epstein–Barr infectious mononucleosis: prospective experience

**DOI:** 10.1007/s15010-022-01932-6

**Published:** 2022-10-12

**Authors:** Gabriel Bronz, Benedetto P. E. S. M. Zanetti, Mario G. Bianchetti, Gregorio P. Milani, Sebastiano A. G. Lava, Thomas J. Neuhaus, Anne Witschi, Lisa Kottanattu

**Affiliations:** 1 Pediatric Institute of Southern Switzerland, Ente Ospedaliero Cantonale, EOC, Bellinzona, Switzerland; 2 Faculty of Biomedical Sciences, Università della Svizzera Italiana, USI, Lugano, Switzerland; 3 Pediatric Unit, Fondazione IRCCS Ca’ Granda Ospedale Maggiore Policlinico, Milan, Italy; 4 Department of Clinical Sciences and Community Health, Università degli Studi di Milano, Milan, Italy; 5 Pediatric Cardiology Unit, Department of Pediatrics, Centre Hospitalier Universitaire Vaudois and University of Lausanne, CHUV, Lausanne, Switzerland; 6 Department of Paediatric Cardiology, Heart Failure and Transplantation, Great Ormond Street Hospital, London, UK; 7 Children’s Hospital of Lucerne, Cantonal Hospital Lucerne, LUKS, Lucerne, Switzerland; 8 Medbase Bern Bahnhof, Bern, Switzerland

**Keywords:** Epstein–Barr infectious mononucleosis, Hoagland sign, Upper eyelid edema, Upper eyelid swelling

## Abstract

**Background:**

The typical presentation of Epstein–Barr virus infectious mononucleosis includes fever, pharyngitis, measles-like rash, jaundice, and enlarged lymph nodes, liver, or spleen. A painless bilateral swelling of the upper eyelid, sometimes with drooping of the lateral aspect, may also occur. This sign, referred to as Hoagland sign, is not or only marginally mentioned in reviews and textbooks.

**Methods:**

Between 2019 and 2021, two of us evaluated all subjects with a positive acute Epstein–Barr virus serology for the typical signs of mononucleosis and for the possible existence of the Hoagland sign.

**Results:**

During the mentioned period, the diagnosis of mononucleosis was made in 26 (14 females and 12 males) subjects aged from 9.0 to 33 years. The initial presentation included fever in 24, enlarged cervical lymph nodes in 23, pharyngitis in 21, a palpable liver in 7, a palpable spleen in 7, jaundice in 2, and a measles-like rash in 2 cases. The Hoagland sign was noted in 14 cases. Patients with and without Hoagland sign did not significantly differ with respect to age and sex.

**Conclusions:**

The Hoagland sign is an easily identifiable clinical sign that is common and likely helpful early in the course of Epstein–Barr virus infectious mononucleosis. There is a need to expand awareness of this sign among physicians.

## Introduction

Primary Epstein–Barr virus infectious mononucleosis, subsequently referred to as mononucleosis, classically affects children and young adults [1, 2]. The clinical signs at presentation include fever, pharyngitis, measles-like rash, jaundice, and enlarged posterior cervical lymph nodes, liver or spleen [1–3]. In previously healthy subjects, the condition typically resolves over a period of weeks [1–3].

**Fig. 1:**
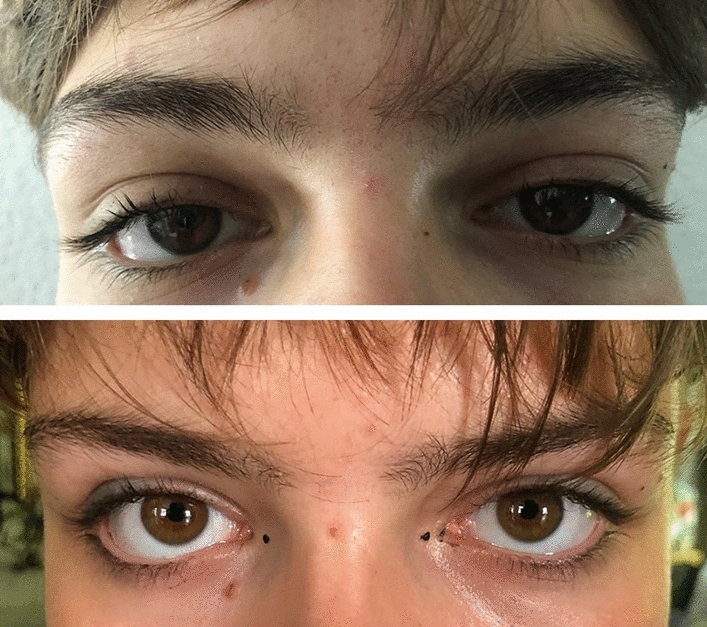
An 18-year-old female adolescent with a serologically proven primary Epstein–Barr virus infectious mononucleosis. At presentation (upper panel) there is a painless bilateral swelling of the upper eyelid, sometimes with drooping of the lateral aspect and narrowing of the ocular aperture (“sleepy face” appearance). The finding normalizes in 2 weeks (lower panel). Permission to publish the images was obtained from the patient

A painless bilateral swelling of the upper eyelid, sometimes with drooping of the lateral aspect, which often results in a narrower ocular aperture and, subsequently, in a “sleepy face” appearance, may be observed in mononucleosis (Fig. 1). This phenomenon, first reported in 1952 by Robert J. Hoagland (1909–2007), is nowadays mostly referred to as Hoagland sign [4]. Reviews and textbooks do not or only marginally refer to the Hoagland sign in mononucleosis and its prevalence is unknown. Hence, we would like to present our prospective experience [1–3].

## Methods

Between 2019 and 2021, all subjects with a positive heterophil antibody test or a specific acute Epstein–Barr virus serology [2, 3] were evaluated by three of the authors both for the typical clinical signs of mononucleosis (axillary body temperature ≥ 38.0 °C; pharyngeal erythema; measles-like rash; yellowish conjunctival discoloration, subsequently referred to as jaundice; posterior cervical lymph nodes ≥ 1.0 cm in diameter; palpable liver edge below the costal margin in the midclavicular line; palpable spleen) and for the possible existence of the Hoagland sign. The lymphocyte to total white blood cell count in peripheral blood was also assessed: a ratio of > 0.35 is considered highly specific for mononucleosis [5].

Continuous data are presented as median and interquartile range and were analyzed by means of the Mann–Whitney–Wilcoxon test [6]. Categorical data are presented as frequency and were analyzed using the Fisher exact test [6]. Significance was set at *p* < 0.05.

## Results

During the mentioned period, the diagnosis of mononucleosis was made in 26 (14 females and 12 males) subjects aged from 9.0 to 33, median 19 years. The diagnosis was supported by a positive heterophile antibody test in 12 and by a specific acute serology response (immunoglobulin M against the Epstein–Barr viral capsid) in 14 cases. The lymphocyte to total white blood cell count, assessed in 23 cases, was found to be > 0.35 in 19 of them.

**Fig. 2:**
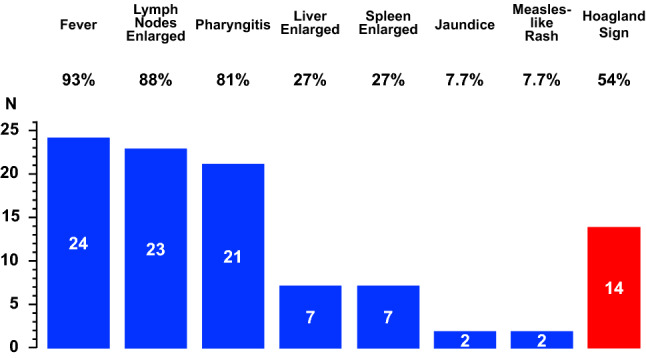
Initial clinical features in 26 patients (14 females and 12 males aged from 9.0 to 33, median 19 years) with acute Epstein–Barr virus infectious mononucleosis

The initial presentation included fever in 24, enlarged posterior cervical lymph nodes in 23, pharyngitis in 21, a palpable liver in 7, a palpable spleen in 7, jaundice in 2, and a measles-like rash in 2 cases. The Hoagland sign was noted in 14 cases (Fig. 2). Patients with (4 males and 10 females, 18 [16–23] years of age) and without (8 males and 4 females, 23 [18–25] years of age) Hoagland sign did not significantly differ with respect to age and sex.

## Discussion

The presentation of mononucleosis may be heterogenous and, therefore, challenging [1–3]. The initial diagnosis of streptococcal pharyngitis (especially in throat carriers of this germ) or that of non-specific pharyngitis are often made. Furthermore, a leukemia or a lymphoma are occasionally suspected. Hence, early and rapid diagnosis is crucial [1–3]. The results of the present prospective experience point out that the Hoagland sign adds to the list of the common and characteristic features of mononucleosis and is likely more frequent than enlarged liver, enlarged spleen, jaundice or rash.

Our results are supported by the seminal observations of Hoagland in 1952 [4]: upper eyelid puffiness was found early in the course of illness in 2 out of 39 (3.6%) soldiers (likely predominantly males) with mononucleosis included in a retrospective case series and, subsequently in 19 out of 56 (34%) soldiers included in a prospective case series. In the present experience, cases with and without Hoagland sign did not significantly differ with respect to age and sex. However, the sign tended to be more common among females than among males (the tendency, however, failed to be statistically significant).

Eyelid features resembling the Hoagland sign occur following trauma or insect sting, in infections of the palpebral region, in many allergic conditions and in diseases that cause fluid retention including among others some kidney diseases [7]. Medication has also been implicated as a cause of eyelid swelling [7]. Taking a careful history and performing a thorough physical examination are essential to distinguish the Hoagland sign from the mentioned causes of eyelid swelling [7].

At least two different mechanisms might underly the development of the Hoagland sign in mononucleosis. An infiltrate of lymphocytes in the periorbital tissue has been occasionally documented [8]. On the other hand, in patients with the Hoagland sign, there is often drooping of the lateral aspect of the upper eyelid, a feature termed S-sign that is usually associated with an enlarged lacrimal gland. In various cases of mononucleosis presenting with an upper lid swelling, orbital imaging revealed an enlarged lacrimal gland [9, 10].

The main limitations of this experience relate to the small number of included cases, who were examined by no more than three physicians, and to the failure to perform a periorbital ultrasound examination. Furthermore, in many cases, the diagnosis of Epstein–Barr mononucleosis was not supported by a specific acute serology response but uniquely by a heterophile antibody test. However, given that a falsely positive heterophile antibody tests is very uncommon, a positive result almost always indicates Epstein–Barr infection.

## Conclusion

The present experience points out that the Hoagland sign is an easily identifiable clinical sign that is common and likely helpful early in the course of mononucleosis. There is a need to expand awareness of this sign among physicians, especially infectiologists and the general practitioners.

## Data Availability

Data are available on request from first author.
